# Psychological Impact of COVID-19 on College Students After School Reopening: A Cross-Sectional Study Based on Machine Learning

**DOI:** 10.3389/fpsyg.2021.641806

**Published:** 2021-04-29

**Authors:** Ziyuan Ren, Yaodong Xin, Junpeng Ge, Zheng Zhao, Dexiang Liu, Roger C. M. Ho, Cyrus S. H. Ho

**Affiliations:** ^1^Department of Medical Psychology and Ethics, School of Basic Medicine Sciences, Cheeloo College of Medicine, Shandong University, Jinan, China; ^2^School of Statistics and Management, Shanghai University of Finance and Economics, Shanghai, China; ^3^School of Biology Engineering, Shandong Jianzhu University, Jinan, China; ^4^Department of Psychological Medicine, Yong Loo Lin School of Medicine, National University of Singapore, Singapore, Singapore

**Keywords:** COVID-19, anxiety, depression, college student, machine learning

## Abstract

COVID-19, the most severe public health problem to occur in the past 10 years, has greatly impacted people's mental health. Colleges in China have reopened, and how to prevent college students from suffering secondary damage due to school reopening remains elusive. This cross-sectional study was aimed to evaluate the psychological impact of COVID-19 after school reopening and explore via machine learning the factors that influence anxiety and depression among students. Among the 478 valid online questionnaires collected between September 14th and September 20th, 74 (15.5%) showed symptoms of anxiety (by the Self-Rating Anxiety Scale), and 155 (32.4%) showed symptoms of depression (by Patient Health Questionnaire-9). Descriptive analysis of basic personal characteristics indicated that students at a higher grade, having relatives or friends who have been infected, fearing being infected, and having a pessimistic attitude to COVID-19 easily experience anxiety or depression. The Synthetic Minority Oversampling Technique (SMOTE) was utilized to counteract the imbalance of retrieved data. The Akaike Information Criterion (AIC) and multivariate logistic regression were performed to explore significant influence factors. The results indicate that exercise frequency, alcohol use, school reopening, having relatives or friends who have been infected, self-quarantine, quarantine of classmates, taking temperature routinely, wearing masks routinely, sleep quality, retaining holiday, availability of package delivery, take-out availability, lockdown restriction, several areas in school closed due to COVID-19, living conditions in the school, taking the final examinations after school reopening, and the degree to which family economic status is influenced by COVID-19 are the primary influence factors for anxiety or depression. To evaluate the effect of our model, we used 5-fold cross-validation, and the average area under the curve (AUC) values of the receiver operating characteristic (ROC) curves of anxiety and depression on the test set reached 0.885 and 0.806, respectively. To conclude, we examined the presence of anxiety and depression symptoms among Chinese college students after school reopening and explored many factors influencing students' mental health, providing reasonable school management suggestions.

## Introduction

COVID-19 (coronavirus disease 2019), the most severe public health problem to occur in the past 10 years, has dramatically impacted the medical health service systems worldwide, causing 57,882,183 confirmed cases and 1,377,395 confirmed deaths up to 22 November 2020 (World Health Organization, 2020). It was first discovered in Wuhan, the Hubei province's capital city, China, and rapidly spread to other regions (Guan et al., 2020a,b). Indeed, after strict regulations were administrated across China, including quarantine, mask-wearing, large-scale nucleic acid assay, etc., the situation of COVID-19 in China significantly improved (Tang et al., 2020; Tu et al., 2020). From 19 November to 21 November 2020, the number of new cases discovered in China was 11 (National Health Commission of the People's Republic of China, 2020). However, more psychologists find that psychological problems, especially post-traumatic anxiety and depression, also matter after this dreadful disaster (Mazza et al., 2020; Pappa et al., 2020; Shader, 2020; Vindegaard and Benros, 2020).

Due to the severity of COVID-19 in the first half of this year, universities across China were closed, and all the students stayed at home and took online courses. In May and June, several universities in the so-called low-risk area reopened. Their students came back to attend their final exams, while most universities did not reopen until September 2020 (Ministry of Education of the People's Republic of China, 2020; People's Daily, 2020). Additionally, strict regulations were announced to protect college students from being infected (Ministry of Education of the People's Republic of China, 2020). For example, students must wear masks and accept temperature testing before they can come into the classroom. Besides, delivery services and free entry to the campus are no longer allowed.

Even before the COVID-19, college students are susceptible to mental health challenges facing unprecedented levels of distress, and early adulthood is one of the peak periods for many mental disorders (de Girolamo et al., 2012). Seventy-five percent of patients with mental health disorders had the first onset before 25 (Kessler et al., 2007). In China, it is reported that the prevalence of suicidal ideation was 9.2% among college students in Jilin province, 2019, and the two most significant risk factors were being a senior and family relationship (Wang et al., 2019). College students play a crucial role in the development of a country. Therefore, with media attention on the college campus, the high rates of mental disorder prevalence have become a growing public health problem in many countries. During the COVID-19, young adults and college students faced more mental challenges, including academic pressure, employment pressure, and family pressure. Many previous studies demonstrated that COVID-19 has led to profound mental and behavioral changes among college students (Alemany-Arrebola et al., 2020; Huckins et al., 2020; Ma et al., 2020; Zhai and Du, 2020). Cao et al. performed a cross-sectional study in China and found that 0.9% of the respondents were experiencing severe anxiety, 2.7% were experiencing moderate anxiety, and 21.3% were experiencing mild anxiety (Cao et al., 2020). Similarly, college students' anxiety and depression rates during the early stages of COVID-19 were not optimistic in the United States (Kecojevic et al., 2020) and Bangladesh (Khan et al., 2020). It is necessary and urgent to improve college students' mental status, and any exacerbation due to strict school regulations after school reopening is unacceptable (Giannopoulou et al., 2020; Kalok et al., 2020).

Whether universities should implement strict regulations remains controversial (Beijing News, 2020). Some think it is irrational to sacrifice students' mental health to prevent COVID-19 transmission. Others believe the strict regulation will not cause mental damage to college students. COVID-19 will not be the last pandemic, so it is of great significance to accumulate experience to avoid severe damage to college students' mental health during the next pandemic event. However, no published articles have investigated the current psychological status of students under these regulations. To this end, we conducted this cross-sectional study via an online questionnaire to ascertain the psychological impact after school reopening on students among five universities across China. Further, we performed machine learning to screen out risk and protective factors that influence the college students' mental health status, including school regulation, family situation, and personal living styles. These influence factors may provide some sensible advice for the school administrative department to prevent college students from mental diseases.

## Materials and Methods

### Ethics Statement

This cross-sectional study was approved by the Ethics Committee of Shandong University as a human-involved study with the serial number ECSBMSSDU2020-1-056. The Declaration of Helsinki was strictly followed, and no identifying information was collected. This cross-sectional study's data collection was conducted between September 14th and September 20th, mainly among five universities across China: Shandong University, Shandong Normal University, Qingdao University (Qingdao, Shandong province), Shanghai Tech University, and Shanghai University of Finance and Economics (Shanghai). Because of COVID-19, an online anonymous questionnaire website (www.wenjuan.com) was used. Therefore, no formal consent was acquired. All participants voluntarily ticked off the informed consent in the first item.

### Participants

Among the 548 invited subjects, 508 subjects were invited to complete the questionnaire by the investigators, and 40 were invited by a free open access online questionnaire distribution platform, www.wjx.cn. Among all the 548 retrieved questionnaires, 478 were valid to study further. Two retrieved questionnaires left blanks, 66 left obvious and invalid options, and 2 had an IP address outside China.

### Designed Questionnaire

#### Basic Personal Characteristics

Two sets of basic personal characteristics were listed in the questionnaire: demographic characteristics and personal perspectives on COVID-19. Demographic characteristics included gender, major, grade, and family location. Personal perspectives on COVID-19 included fear of being infected, attitude to COVID-19, history of psychological counseling, and need for psychological counseling. In this study, “psychological counseling” means college students received psychological counseling from their school. In China, the impact of the COVID-19 on college students' mental health has drawn public attention. After the school reopening, the university might provide psychological counseling for all students. The detailed options for each question are presented in Table 1.

**Table 1:** Descriptive analysis of basic characteristics.

**Variable (*n* = 478)**	**All participants (%)**		**Anxiety**				***P-value***	**Depression**				***P-value***
			**NO**		**YES**			**NO**		**YES**		
Gender												
Male	205	42.9%	168	82.0%	37	18.0%	0.18	140	68.3%	65	31.7%	0.77
Female	273	57.1%	236	86.4%	37	13.6%	Chi-square	183	67.0%	90	33.0%	Chi-square
Major												
Medicine/Biology	100	20.9%	87	87.0%	13	13.0%	0.64	63	63.0%	37	37.0%	0.64
Psychology	151	31.6%	129	85.4%	22	14.6%	Chi-square	107	70.9%	44	29.1%	Chi-square
Science/Engineering	59	12.3%	51	86.4%	9	15.3%		40	67.8%	19	32.2%	
Others	168	35.1%	137	81.5%	31	18.5%		113	67.3%	55	32.7%	
Grade												
Fresher	110	23.0%	100	90.9%	10	9.1%	0.03	82	74.5%	28	25.5%	0.35
Sophomore	182	38.1%	154	84.6%	28	15.4%	Chi-square	119	65.4%	63	34.6%	Chi-square
Junior	139	29.1%	116	83.5%	23	16.5%		92	66.2%	47	33.8%	
Senior (4th/5th) and above	47	9.8%	34	72.3%	13	27.7%		30	63.8%	17	36.2%	
Family location												
Rural/County areas	227	47.5%	194	85.5%	33	14.5%	0.59	158	69.6%	69	30.4%	0.37
City	251	52.5%	210	83.7%	41	16.3%	Chi-square	165	65.7%	86	34.3%	Chi-square
Fear of being infected												
Very low	186	38.9%	169	90.9%	17	9.1%	<0.001	140	75.3%	46	24.7%	<0.001
Low	152	31.8%	131	86.2%	21	13.8%	Somer's d	100	65.8%	52	34.2%	Somer's d
Medium	92	19.2%	71	77.2%	21	22.8%		55	59.8%	37	40.2%	
High	33	6.9%	22	66.7%	11	33.3%		20	60.6%	13	39.4%	
Very high	15	3.1%	11	73.3%	4	26.7%		8	53.3%	7	46.7%	
Attitude to COVID-19												
Very pessimistic	30	6.3%	24	80.0%	6	20.0%	<0.001	17	56.7%	13	43.3%	<0.001
Pessimistic	36	7.5%	25	69.4%	11	30.6%	Somer's d	21	58.3%	15	41.7%	Somer's d
Medium	100	20.9%	78	78.0%	22	22.0%		54	54.0%	46	46.0%	
Optimistic	120	25.1%	102	85.0%	18	15.0%		78	65.0%	42	35.0%	
Very optimistic	192	40.2%	175	91.1%	17	8.9%		153	79.7%	39	20.3%	
History of counseling												
Yes	215	45.0%	183	85.1%	32	14.9%	0.74	154	71.6%	61	28.4%	0.09
No	263	55.0%	221	84.0%	42	16.0%	Chi-square	169	64.3%	94	35.7%	Chi-square
Need for counseling												
Yes	60	12.6%	32	53.3%	28	46.7%		26	43.3%	34	56.7%	
No	352	73.6%	321	91.2%	31	8.8%		266	75.6%	86	24.4%	
Not sure	66	13.8%	51	77.3%	15	22.7%		31	47.0%	35	53.0%	

*Counseling here means psychological counseling received from school or from others. Somer's d was used to measure the consistency of ordered categories. Chi-square test was used to measure correlations between unordered categories*.

#### Assessment of Anxiety and Depression

The classic Zung's Self-rating Anxiety Scale (SAS) was used to evaluate the participants' anxiety degree (Zung, 1971); it includes 20 self-reported items about moods, sleep, sense of pain, etc. After the standardized scoring algorithm, four anxiety degree grades were defined. A score of 20–49 was considered as no anxiety, 50–59 as mild anxiety, 60–69 as moderate anxiety, and 70–80 as severe anxiety. Zhou et al. demonstrated that the reliability and validity of SAS applied in Chinese college students were acceptable. The criterion defining normal/mild/moderate/severe anxiety was suitable for Chinese (Yongan, 2012). The Cronbach's alpha was 0.906 for SAS in the current study.

The Patient Health Questionnaire-9 (PHQ-9) was used to evaluate the depression degree (Kroenke et al., 2001); it includes nine self-reported items with a score of 0–4 for each question. After the standardized scoring algorithm, five depression degree grades were defined. A score of 0–4 was considered as no depression, 5–9 as mild depression, 10–14 as moderate depression, 15–19 as moderate to severe depression, and 20–27 as severe depression. This scale has been confirmed to be a reliable and valid tool in assessing mental health in Chinese adolescents (Xingchen et al., 2014). In this study, the Cronbach's alpha for PHQ-9 was 0.918.

#### Influence Factor Selection

All the influence factors included in our questionnaire were acquired from a 20-subject pre-survey. We randomly invited 20 subjects included in the pre-survey from Shandong University. Two investigators (Y.X and J.P) searched the Pubmed and retrieved 30 potential influence factors. We asked 20 subjects whether they agreed a certain factor might significantly influence students' psychological condition. Therefore, we selected those factors which are agreed by most subjects (>10/20). After considering all the retrieved influence factors comprehensively, three sets of questions were included in our questionnaire: school regulations, family situation, and personal living style. School regulations mean the regulations announced during COVID-19 to prevent disease transmission across schools, including school reopening, routine mask-wearing, routine temperature-taking, several areas in school being closed due to COVID-19, final examinations being taken after school reopening, retaining holiday (whether to shorten or cancel the holiday after school reopening), availability of package delivery (after school reopening), take-out food availability (after school reopening), lockdown restriction (whether to allow free access to campus after school reopening), quarantine of classmates (after school reopening), and self-quarantine after school reopening. The “retaining holiday (whether to shorten or cancel the holiday after school reopening)” here refers to the fact that a number of universities in China have shortened or canceled some holidays in order to minimize the students' total in-school time after school reopening during COVID-19. “Holiday” here includes weekends and statutory holidays in China. The family situation means the impact of COVID-19 on family, including having relatives or friends who have been infected and the degree to which family economic status was influenced by COVID-19. The personal living style includes exercise frequency, alcohol use, sleep quality, and satisfaction with school living conditions. The detailed options for each question are presented in Table 2.

**Table 2:** Descriptive statistics of influence factors.

**Influence factors**	**Anxiety**					**Depression**				
	**NO**		**Yes**		***P-value***	**No**		**Yes**		***P-value***
***School regulation***										
School reopening										
Yes	397	85.40%	68	14.60%	*F-P*	314	67.50%	151	32.50%	*F-P*
No	7	53.80%	6	46.20%	0.008	9	69.20%	4	30.80%	0.58
Wearing masks routinely										
Yes	246	84.80%	44	15.20%	*C-P*	202	69.70%	88	30.30%	*C-P*
No	158	84.00%	30	16.00%	0.817	121	64.40%	67	35.60%	0.227
Taking temperature routinely										
Yes	339	84.30%	63	15.70%	*C-P*	267	66.40%	135	33.60%	*C-P*
No	65	85.50%	11	14.50%	0.791	56	73.70%	20	26.30%	0.215
Several areas in school closed due to COVID-19 (136 reported not sure)										
Yes	135	82.80%	28	17.20%	*C-P*	101	62.00%	62	38.00%	*C-P*
No	149	83.20%	30	16.80%	0.918	132	73.70%	47	26.30%	0.02
Taking the final examination after school reopening										
Yes	237	86.80%	36	13.20%	*C-P*	190	69.60%	83	30.40%	*C-P*
No	167	81.50%	38	18.50%	0.11	133	64.90%	72	35.10%	0.275
Retaining holiday (119 reported not sure)										
Yes	100	86.20%	16	13.80%	*C-P*	80	69.00%	36	31.00%	*C-P*
No	200	82.30%	43	17.70%	0.351	165	67.90%	78	32.10%	0.839
Availability of package delivery (28 reported not sure)										
Yes	313	86.50%	49	13.50%	*C-P*	244	67.40%	118	32.60%	*C-P*
No	68	77.30%	20	22.70%	0.032	60	68.20%	28	31.80%	0.889
Take-out availability (31 reported not sure)										
Yes	41	67.20%	20	32.80%	*C-P*	36	59.00%	25	41.00%	*C-P*
No	335	86.80%	51	13.20%	<0.001	264	68.40%	122	31.60%	0.147
Lockdown restriction (19 reported not sure)										
Yes	364	85.60%	61	14.40%	*C-P*	287	67.50%	138	32.50%	*C-P*
No	23	67.60%	11	32.40%	0.005	22	64.70%	12	35.30%	0.736
Quarantine of classmates										
Yes	51	75.00%	17	25.00%	*C-P*	36	52.90%	32	47.10%	*C-P*
No	353	86.10%	57	13.90%	0.019	287	70.00%	123	30.00%	0.005
Self-quarantine after school reopening										
Yes	9	47.40%	10	52.60%	*C-P*	7	36.80%	12	63.20%	*C-P*
No	395	86.10%	64	13.90%	<0.001	316	68.80%	143	31.20%	0.003
***Family situation***										
Having relatives or friends who have been infected										
Yes	4	50.00%	4	50.00%	*F-P*	2	25.00%	6	75.00%	*F-P*
No	400	85.10%	70	14.90%	0.023	321	68.30%	149	31.70%	0.016
The degree of family economic status influenced by COVID-19 (1 for little, 5 for much)										
1	136	94.40%	8	5.60%		110	76.40%	34	23.60%	
2	87	74.40%	30	25.60%	S	71	60.70%	46	39.30%	S
3	119	88.10%	16	11.90%	0.066	90	66.70%	45	33.30%	0.06
4	35	70.00%	15	30.00%	*p*	31	62.00%	19	38.00%	*p*
5	27	84.40%	5	15.60%	0.004	21	65.60%	11	34.40%	0.053
***Personal living style***										
Exercise frequency (past 1 week)										
nearly 0 time	87	82.10%	19	17.90%		62	58.50%	44	41.50%	
1 time	79	78.20%	22	21.80%	S	64	63.40%	37	36.60%	S
2 times	89	88.10%	12	11.90%	−0.046	69	68.30%	32	31.70%	−0.092
3 times	45	83.30%	9	16.70%	*p*	41	75.90%	13	24.10%	*p*
more than 4 times	104	89.70%	12	10.30%	0.045	87	75.00%	29	25.00%	0.002
Alcohol use (past 2 week)										
nearly 0 time	314	88.00%	43	12.00%		251	70.30%	106	29.70%	
1 time	52	76.50%	16	23.50%	S	42	61.80%	26	38.20%	S
2 times	18	66.70%	9	33.30%	0.124	16	59.30%	11	40.70%	0.102
3 times	9	64.30%	5	35.70%	*p*	7	50.00%	7	50.00%	*p*
more than 4 times	11	91.70%	1	8.30%	0.002	7	58.30%	5	41.70%	0.03
Self-rating sleep quality (1 for very poor, 5 for very good)										
1	39	81.30%	9	18.80%		29	60.40%	19	39.60%	
2	58	73.40%	21	26.60%	S	45	57.00%	34	43.00%	S
3	94	81.70%	21	18.30%	−0.089	70	60.90%	45	39.10%	−0.147
4	96	86.50%	15	13.50%	*p*	69	62.20%	42	37.80%	*p*
5	117	93.60%	8	6.40%	<0.001	110	88.00%	15	12.00%	<0.001
Satisfaction of living conditions in school (1 for very unsatisfied, 5 for very satisfied)										
1	52	85.20%	9	14.80%		43	70.50%	18	29.50%	
2	83	80.60%	20	19.40%	S	69	67.00%	34	33.00%	S
3	110	78.00%	31	22.00%	−0.059	82	58.20%	59	41.80%	−0.043
4	81	89.00%	10	11.00%	*p*	64	70.30%	27	29.70%	*p*
5	78	95.10%	4	4.90%	0.005	65	79.30%	17	20.70%	0.141

*F-P, P-value of Fisher's test; C-P, P-value of the Chi-square test; S, Somer's d; p, P-value of Somer's d*.

### Statistics

Correlation analysis was performed for univariate analysis to primarily determine whether factors have relations with students' anxiety or depression conditions in a descriptive view. More specifically, for independent variables from two categories or multiple unordered categories, we used the chi-square test. For the cells in which the samples numbered <5, we used Fisher's exact test. For explanatory variables from multiple ordered categories, we used Somer's d to measure the consistency between the two (that is, whether the two tend to move in the same or opposite directions).

Logistic regression was performed for multivariate analysis to determine the association between a particular factor and students' psychological status quantitively, other factors being equal. First, we divided the samples into five equal parts in order to perform 5-fold cross-validation. Second, the Synthetic Minority Oversampling Technique (SMOTE) was performed on the training dataset to preprocess the retrieved data. As is acknowledged to all, the number of positive and negative samples of medical data is often uneven, which could strongly affect the effectiveness of the Logistic regression model. SMOTE is an oversampling algorithm that generates extra samples based on the original dataset. By setting a specific scale, SMOTE can make the dataset balanced using methods of oversampling. The Akaike Information Criterion (AIC) and binary logistic regression were performed to explore significant influence factors. The accuracy, sensitivity (recall), specificity (accuracy = (TP + TN)/(TP + TN + FP + FN), sensitivity = TP/(TP + FN), specificity = TN/(TN + FP), where TP is correct positive assignments, TN is correct negative assignments, FP is incorrect positive assignments, and FN is incorrect negative assignments) and area under the curve (AUC) values of the receiver operating characteristic (ROC) curves were used to evaluate the machine learning model.

Data collection and descriptive analysis were performed using Excel 2016 (Microsoft, Washington, D.C., US). Univariate analyses were performed using IBM SPSS Statistics 24.0 (International Business Machines Corporation, Armonk, NY, USA). Multivariate analyses were performed using the R language(R Core Team, 2020). A *P*-value <0.05 was considered statistically significant.

## Results

### Presence of Anxiety and Depression Symptoms

This survey's response variables were anxiety and depression evaluated by SAS and PHQ-9, respectively. Among all the 478 valid subjects, 74 (15.5%) showed symptoms of anxiety (among which 4 (0.8%) showed severe anxiety, 15 (3.1%) showed moderate anxiety, and 55 (11.5%) showed mild anxiety). Besides, 155 (32.4%) showed symptoms of depression (among which 9 (1.9%) showed severe depression, 26 (5.4%) showed moderate to severe depression, 46 (9.6%) showed moderate depression, and 74 (15.5%) showed mild depression). We divided subjects into two sets—anxiety (depression) and normal (normal)—for further influence factor exploration.

### Descriptive Analysis of Basic Personal Characteristics

The basic personal characteristics of the 478 valid subjects are displayed in Table 1. College students at a higher grade, fear being infected, and have a more pessimistic attitude to COVID-19 were more likely to report anxiety or depression, while gender, major, and family location did not significantly differ. After comparing 215 (45%) subjects who have received psychological counseling with the other students who did not, no significant difference in the presence of expression/anxiety symptoms was observed for students who had received counseling (*p* = 0.74 for anxiety, and *p* = 0.09 for depression) compared with the control group, which means that the effect of current psychological counseling is limited. Besides, 60 (12.6%) students reported that they need psychological counseling. Notably, 66 (13.8%) students reported that they had no idea whether they need professional psychological counseling; the presence of anxiety was 22.7%, and that of depression was 53% in this group, which is nearly three times the presence of anxiety and two times the presence of depression observed in the group of students who reported that they did not need counseling. Therefore, the group of students who are not sure whether they need psychological counseling appears particularly vulnerable for experiencing clinically significant depression or anxiety.

### Descriptive Analysis of Influence Factors

The characteristics of the 17 influence factors in our questionnaires are displayed in Table 2. In the set of school regulations, school reopening, several areas in schools being closed due to COVID-19, lockdown restriction, and availability of package delivery alleviated college students' anxiety or depression. A significant relationship between depression or anxiety and take-out availability, quarantine of classmates, and self-quarantine after school reopening was found (*p* < 0.05). Other risk factors were of no statistical significance (*p* > 0.05). In terms of family situation, having relatives or friends who have been infected and the family economic status being influenced to a strong degree by COVID-19 were significantly related to psychological problems. Finally, in personal living style, students who exercise more, drink less, sleep better, and are satisfied with their living conditions reported a healthier psychological state.

### Construction of the Logistic Regression Model

**Figure 1:**
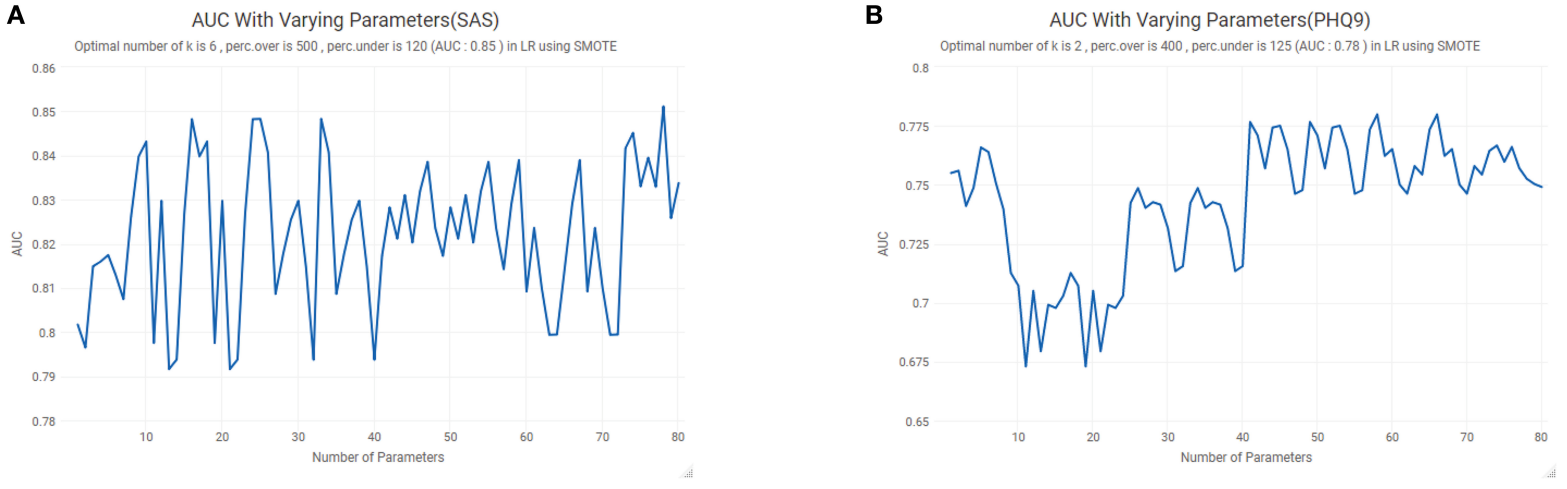
The Synthetic Minority Oversampling Technique (SMOTE): **(A)** anxiety; **(B)** depression. AUC, area under the curve.

SMOTE was performed to counteract the imbalance of the retrieved data (15.5% of subjects showed a symptom of anxiety, and 32.4% of subjects show a symptom of depression). When using SMOTE, we need to determine three parameters: *k*, perc.over, and perc.under. *k* represents the number of nearest neighbors used to generate new instances of the minority classes. perc.over decides how many additional cases to generate from the minority classes (known as oversampling). perc.under decides how many extra cases from the majority classes are selected for each case generated from the minority class (known as under-sampling). As shown in Figure 1A, for the SAS dataset, when *k* = 6, perc.over = 500, and perc.under = 120, the AUC reaches the maximum. Similarly, for the PHQ9 dataset, when *k* = 2, perc.over = 400, and perc.under = 125, the AUC reaches the maximum (Figure 1B). Since we had divided the dataset into five equal parts, we selected one part as the test set each time and using SMOTE to process the other four groups. A new dataset was thus utilized in the construction of the logistic regression model for anxiety and depression.

The AIC (Table 3) was applied to select proper influence factors for inclusion in the logistic regression model. Among the 17 influence factors acquired from a 20-subject pre-survey, 13 factors (taking temperature routinely, retaining holiday, self-rated sleep quality, taking the final examination after school reopening, lockdown restriction, exercise frequency, quarantine of classmates, take-out availability, alcohol use, availability of package delivery, school reopening, self-quarantine after school reopening, and the degree to which family economic status is influenced by COVID-19) were extracted according to the AIC for anxiety, while 15 factors (routinely wearing masks, having relatives or friends who have been infected, satisfaction with living conditions in the school, taking temperature routinely, several areas in school being closed due to COVID-19, self-rated sleep quality, taking the final examination after school reopening, lockdown restriction, exercise frequency, quarantine of classmates, take-out availability, alcohol use, availability of package delivery, self-quarantine after school reopening, and the degree to which family economic status is influenced by COVID-19) were extracted for depression.

**Table 3:** The Akaike Information Criterion (AIC) values of the 17 influence factors for anxiety and depression.

**In/Out**	**Influence factor**	**Df**	**Deviance**	**AIC**
**Anxiety**				
	<none>		447.52	507.52
Out	wearing masks routinely	1	446.65	508.65
Out	having relatives or friends who have been infected	1	447.49	509.49
Out	satisfaction of living conditions in school (1 for very unsatisfied, 5 for very satisfied)	4	441.6	509.6
In	taking temperature routinely	1	451.68	509.68
Out	several areas in school closed due to COVID-19	1	446.72	510.72
In	retaining holiday	1	456.94	512.94
In	self-rating sleep quality (1 for very poor, 5 for very good)	4	463.64	515.64
In	taking final examination after school reopening	1	457.95	515.95
In	lockdown restriction	1	462.73	518.73
In	exercise frequency (past 1 week)	4	472.48	524.48
In	quarantine of classmates	1	467.86	525.86
In	take-out food availability	1	470.69	526.69
In	alcohol use (past 2 week)	4	476.08	528.08
In	availability of package delivery	1	472.56	528.56
In	school reopening	1	473.96	531.96
In	self-quarantine after school reopening	1	476.11	534.11
In	the degree of family economic status influenced by COVID-19 (1 for little, 5 for much)	4	482.45	534.45
**Depression**				
	<none>		1205.8	1275.8
In	taking the final examination after school reopening	1	1208.5	1276.5
Out	retaining holiday	1	1203	1277
In	taking temperature routinely	1	1209.2	1277.2
Out	school reopening	1	1205.8	1277.8
In	several areas in school closed due to COVID-19	1	1212.5	1278.5
In	availability of package delivery	1	1213.7	1279.7
In	exercise frequency (past 1 week)	4	1227	1289
In	the degree of family economic status influenced by COVID-19 (1 for little, 5 for much)	4	1230.2	1292.2
In	take-out food availability	1	1228.2	1294.2
In	satisfaction of living conditions in school (1 for very unsatisfied, 5 for very satisfied)	4	1233.9	1295.9
In	alcohol use (past 2 week)	4	1240.1	1302.1
In	wearing masks routinely	1	1235.3	1303.3
In	lockdown restriction	1	1237.3	1303.3
In	having relatives or friends who have been infected	1	1238.5	1306.5
In	quarantine of classmates	1	1239	1307
In	self-quarantine after school reopening	1	1256.2	1324.2
In	self-rating sleep quality (1 for very poor, 5 for very good)	4	1272.8	1334.8

“*In” means included in the logistic regression model; “Out” means excluded from the logistic regression model; DF, degrees of freedom*.

We used a conventional generalized linear model, Logistic regression, to analyze the data after selecting factors. For each factor, we set the first level as the control group. The results of the logistic regression are presented in Table 4. The binary logistic regression finally extracted 12 significant influence factors (excluding sleep quality from the list of 13 above) for anxiety and 12 (excluding taking temperature routinely, taking the final examination after school reopening, and availability of package delivery from the list of 15 above) for depression (*p* < 0.05).

**Table 4:** Binary logistic regression model.

	**Anxiety**		**Depression**	
	**OR(95% CI)**	***P-value***	**OR(95% CI)**	***P-value***
***School regulation***				
School reopening				
Yes	reference		none	
No	21.99(6.84,70.68)	<0.001		
Wearing masks routinely				
Yes			reference	
No			2.33(1.71,3.17)	<0.001
Taking temperature routinely				
Yes	reference		reference	
No	1.88(1.10,3.23)	0.021	0.7(0.47,1.03)	0.067 (out)
Several areas in school closed due to COVID-19				
Yes	none		reference	
No			0.64(0.45,0.9)	0.011
Taking the final examination after school reopening				
Yes	reference		reference	
No	2.40(1.53,3.79)	<0.001	1.27(0.96,1.7)	0.099 (out)
Retaining holiday				
Yes	reference		none	
No	1.85(1.07,3.21)	0.028		
Availability of package delivery				
Yes	reference		reference	
No	3.53(2.06,6.03)	<0.001	1.13(0.77,1.64)	0.5329 (out)
Tale-out food availability				
Yes	reference		reference	
No	0.21(0.12,0.37)	<0.001	0.39(0.26,0.58)	<0.001
Lockdown restriction				
Yes	reference		reference	
No	4.35(2.33,8.33)	<0.001	2.63(1.67,4.17)	<0.001
Quarantine of classmates				
Yes	reference		reference	
No	0.23(0.12,0.42)	<0.001	0.32(0.22,0.48)	<0.001
Self-quarantine after school reopening				
Yes	reference		reference	
No	0.15(0.07,0.33)	<0.001	0.13(0.07,0.26)	<0.001
***Family situation***				
Having relatives or friends who have been infected				
Yes	none		reference	
No			0.08(0.03,0.24)	<0.001
The degree of family economic status influenced by COVID-19 (1 for little, 5 for much)				
1	reference		reference	
2	4.56(2.31,9.00)	<0.001	1.86(1.23,2.8)	0.003
3	1.00(0.5,2.04)	0.990	1.37(0.92,2.05)	0.124
4	4.7(2.15,10.28)	<0.001	3.28(1.91,5.64)	<0.001
5	4.35(1.69,11.18)	0.002	2.26(1.26,4.06)	0.006
***Personal living style***				
Exercise frequency (past 1 week)				
nearly 0 time	reference		reference	
1 time	1.48(0.80,2.73)	0.215	0.65(0.43,1.01)	0.053
2 times	0.54(0.28,1.03)	0.060	0.75(0.49,1.15)	0.185
3 times	0.90(0.40,2.03)	0.802	0.45(0.26,0.78)	0.004
more than 4 times	0.37(0.18,0.74)	0.005	0.42(0.28,0.63)	<0.001
Alcohol use (past 2 week)				
nearly 0 time	reference		reference	
1 time	1.86(1.06,3.26)	0.029	1.76(1.21,2.56)	0.003
2 times	3.97(1.67,9.44)	0.002	3.79(2.03,7.06)	<0.001
3 times	9.21(1.78,47.58)	0.008	4.47(1.88,10.63)	0.001
more than 4 times	0.15(0.02,1.11)	0.064	1.02(0.37,2.79)	0.975
Self-rating sleep quality (1 for very poor, 5 for very good)				
1	reference		reference	
2	1.73(0.72,4.19)	0.221 (out)	0.73(0.39,1.37)	0.326
3	1.66(0.70,3.94)	0.254	0.78(0.43,1.4)	0.406
4	1.15(0.49,2.67)	0.747	0.66(0.37,1.18)	0.165
5	0.61(0.24,1.54)	0.296	0.17(0.09,0.32)	<0.001
Satisfaction of living conditions in school (1 for very unsatisfied, 5 for very satisfied)				
1	none		reference	
2			2.44(1.31,4.53)	0.005
3			3.32(1.85,5.94)	<0.001
4			1.48(0.79,2.76)	0.217
5			3.04(1.61,5.75)	0.001

*OR, odds ratio; CI, confidence interval; “out” means no statistical significance; “none” means excluded by AIC*.

### Evaluation of the Logistic Regression Model

**Figure 2:**
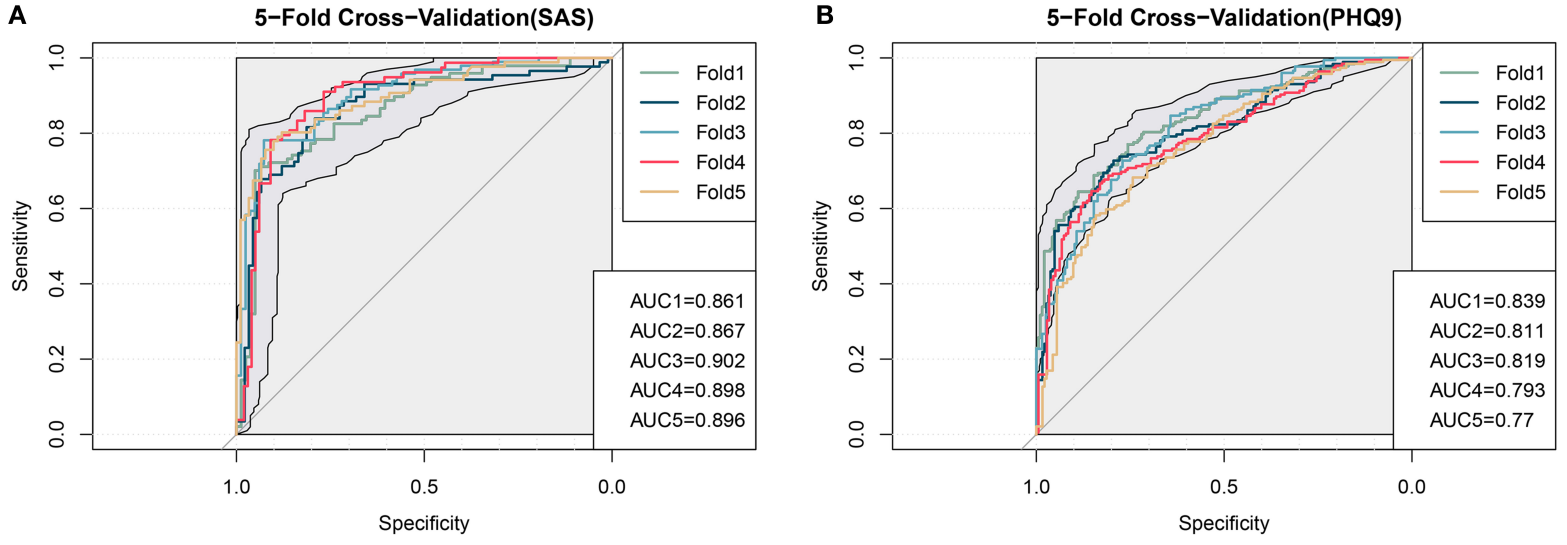
Evaluation of the logistic model by 5-fold cross-validation. **(A)** anxiety; **(B)** depression. AUC, area under the curve.

When applying a logistic regression model, it is crucial to avoid overfitting. To evaluate our model effectively, we first divided the dataset into five equal parts, using four of them to train a regression model. Then, we tested the generated model to see whether there was a significant difference. The ROC curves of 5-fold cross-validation were plotted to evaluate our logistic regression model (Figure 2), where the shadow area represents the 95% confidence interval of the ROC curve. The anxiety model's average accuracy was 81.42%, and that of the depression model was 73.5%. On the test dataset, the average AUC of the anxiety model was 0.885, and that of the depression model was 0.806, which indicates that the predictive power of our models is excellent. The average sensitivity (recall) of the models reached 83.21 and 75.3%, respectively. The average specificity of the models reached 80.38 and 71.80%, respectively. The sensitivity and specificity were both acceptable.

## Discussion

In this study, we performed a cross-sectional survey to investigate the presence of anxiety and depression symptoms among Chinese college students after school reopening and explored a series of factors influencing students' mental health.

First, the descriptive statistics of basic personal characteristics were utilized to test whether these basic characteristics would affect students' emotional status. The result shows that students having relatives or friends who have been infected, fearing being infected, having a more pessimistic attitude toward COVID-19 were more likely to report psychological problems. Moreover, we found that students at a higher grade easily got anxious and depressed. First, senior students must participate in a practicum, which has been demonstrated as a risk factor for stress and anxiety (Cheung et al., 2016). Besides, senior students faced more mental challenges, including greater academic pressure, graduation pressure, etc. Furthermore, due to the pandemic of COVID-19, these pressures were amplified. Of the participants, 60 (12.6%) reported needing psychological help, and 66 (13.8%) reported that they had no idea whether they needed psychological help. These students would be more vulnerable than others for lacking awareness of the importance of psychological health and not getting prompt treatment. Therefore, it is necessary to give students universal mental health education. Besides, among the 478 subjects, 215 (45%) had received psychological counseling from school. However, it had not resulted in a significant improvement of their mental health, which indicates that the effect of current psychological help for college students is limited. Several potential reasons probably cause this. Firstly, the effect of current psychological help, especially online counseling, for college students is limited. Many Chinese university counselors would need training in psychological service. Moreover, it may still require a longer time to observe therapeutic changes of the psychological survey. Universities are essential in dealing with the mental status of college students (Zhai and Du, 2020). It is impractical to provide face-to-face professional psychological counseling to every college student due to financial limitations and psychologists' numbers. Besides selecting students in need by way of the influence factors discussed above, tele-counseling is particularly important in this area. Previous studies have reported that tele-counseling or digital mental health interventions have developmental prospects (Levin et al., 2016, 2017; Lattie et al., 2019). However, some also reported that the current situation of college psychological centers' website effectiveness is compromised. Seidel et al. reported that only half of all 138 analyzed websites provided information about remote counseling. Approximately two-thirds of them had directions for students experiencing a mental health emergency (Seidel et al., 2020). Indeed, how to provide professional psychological help to students in need remains controversial (Lungu and Sun, 2016; Webermann and Murphy, 2020).

Second, univariate and multivariate analysis extracted 17 significant factors influencing college students' mental status. Among these influence factors, four factors of healthier personal lifestyles—higher exercise frequency, lower alcohol use, higher sleep quality, and higher satisfaction with living conditions in the school—were closely related to a lower risk of psychological problems. Several studies in the literature have demonstrated that these influence factors play a crucial role in public mental health (Walsh, 2011; Velten et al., 2018; Oftedal et al., 2019). Notably, it was reported that sleep problems among adolescents and young adults during the COVID-19 epidemic, especially college students, are common and negatively associated with students' projections of trends in COVID-19 (Zhou et al., 2020). Zhang et al. found that sleep problems may mediate the pandemic's impact on mental health (Zhang et al., 2020). Therefore, more attention should be paid to insomnia currently. As for the two family situation influence factors, having relatives or friends who have been infected and unstable family income would cause psychological problems. In terms of the 11 school regulation influence factors, quarantine was a robust factor associated with clinical symptoms of anxiety and depression (Khan et al., 2020; Tang et al., 2020,a; Xin et al., 2020) compared with the control group, suggesting that reducing unnecessary quarantine measures can effectively improve students' mental health. The protective regulations, such as lockdown restriction, were mostly related to better mental health, unexpected before this investigation. This result suggests that college students would rather endure some inconvenience in daily life than be probably infected with the virus, except for some daily necessities, such as delivery and retaining holiday. With the rate of the virus spread slowing down, schools at all levels are reopening. Although the pandemic situation has been much improved, finding the balance between protecting students from coronavirus infection and preventing students from the pressure of delayed schooling, compromised living conditions, and physical health is challenging for policymakers. In this study, we screened the significant influence factors associated with anxiety and depression among college students. According to our findings, several preventive interventions should be mentioned. First, schools should provide professional psychological help for students suffering from COVID-19, especially having relatives or friends who have been infected. Schools should also provide more financial aids for students in poverty during COVID-19. Besides, schools should encourage students to develop healthy lifestyles, including daily exercise and lower alcohol use. Besides, schools should emphasize the importance of sleep, especially in this particular period. Finally, some strict regulations should be applied, such as wearing masks and taking temperature routinely. These measurements would even improve students' mental health. Simultaneously, schools should ensure that some services closely related to students' daily life, such as delivery service, will be maintained.

Previous studies have demonstrated that college students have been suffering extreme mental pressure during this pandemic, and proposed some countermeasures. Chi's study supported interventions promoting resilience, even remotely, to subjects with specific risk factors of developing poor mental health during COVID-19 or other pandemics with social isolation (Chi et al., 2020). Chen et al. found that isolation policy had a complex influence on the symptoms of obsessive–compulsive disorder, fear, hypochondria, depression, and neurasthenia via various factors and introduced a six-step intervention strategy to alleviate young people's psychological problems while in isolation (Chen et al., 2020). Similar studies were performed in many other countries, including the United States (Huckins et al., 2020; Son et al., 2020), Saudi Arabia (Alkhamees et al., 2020), India (Kapasia et al., 2020), Bangladesh (Khan et al., 2020), and Jordan (Naser et al., 2020). Huckins et al. reported that compared with U.S. college students in the prior academic terms, the Winter 2020 term individuals were more sedentary, anxious, and depressed. A wide variety of behaviors, including increased phone usage, decreased physical activity, and fewer locations visited, were associated with fluctuations in COVID-19 news reporting (Huckins et al., 2020).

Some previous studies have also reported some “hub influence factors” that serve as mediators between “ordinary influence factors” and psychological problems. For example, as mentioned above, Zhang et al. utilized R software's mediation package to find that the severity of the COVID-19 outbreak indirectly affects negative emotions by affecting sleep quality (Zhang et al., 2020). Such mediators also include resilience, social support, and coping (Yang et al., 2020a). These “hub factors” could be considered “targets” for psychological interventions, including psychological counsel to strengthen one's resilience and coping ability, social support from friends or family, and even medical intervention. For example, the appropriate application of some hypnotics has been proved to be effective for anxiety and depression patients without significant side effects (Yang et al., 2020b). The “sleep quality” factor derived in our study is one of the previously discovered “hub influence factors” (Zhang et al., 2020). Therefore, we highly suggested that schools encourage students to get enough sleep times and higher sleep quality.

In our study, we did not find any relationship between majors and the mental health of college students. However, many previous studies have reported that the psychological impact of the pandemic on college students majoring in psychology or medicine-related subjects is more significant. Guidotti et al. found that a notable percentage of neuropsychology trainees reported increased personal mental health symptoms (i.e., anxiety/depression; 74/54%) as well as several other personal stressors (Guidotti Breting et al., 2020). Similar situations occurred in Nepal (Khanal et al., 2020; Shrestha, 2020) and China (Xiao et al., 2020). To conclude, as for psychological/medical students, COVID-19 might cause enormous psychological stress. And psychological interventions should be implemented. Besides, more clinical studies should be conducted to prove this point.

This study has some strengths. First, this is the first published article examining college students' mental status after school reopening to the best of our knowledge. Second, the subjects included in this study were from several different schools across China, which increases this study's universality. Third, the application of machine learning algorithms, including SMOTE, AIC, multivariate logistic regression, and ROC curves, is appropriate and reasonable, increasing the study's scientificity and reliability.

This study also has some limitations. First, the sample was relatively small. It is not easy to explore differences among schools. We just used the Chi-square test to prove no significant differences between city areas and rural areas. Second, some infrequent influence factors, which certain subjects mentioned, were not included in the questionnaire. Third, more psychological clinical trials should be performed to discover the susceptibility to other mental problems such as alexithymia (Tang et al., 2020,b) and PTSD after school reopening.

## Conclusion

In this study, we performed a cross-sectional survey to investigate the prevalence of anxiety and depression among Chinese college students after school reopening and explored a series of factors influencing students' mental health. Many studies have demonstrated that college students have been suffering extreme mental pressure during the pandemic. For example, Cao et al. performed a cross-sectional study in China and found that 0.9% of the respondents were experiencing severe anxiety, 2.7% were experiencing moderate anxiety, and 21.3% were experiencing mild anxiety. Also, influence factors and some so-called “hub influence factors” were mentioned. Currently, the pandemic's control status varies worldwide. The situation of COVID-19 in China significantly improved and the school reopens. However, in some other countries, the school might reopen after a period of time. To the best of our knowledge, this is the first published article examining college students' mental status after school reopening. Therefore, we evaluated the school regulation measures based on college students' mental health, which could provide sensitive suggestions for school management worldwide.

## Data Availability Statement

The raw data supporting the conclusions of this article will be made available by the authors, without undue reservation.

## Ethics Statement

The studies involving human participants were reviewed and approved by Ethics Committee of Shandong University. Written informed consent for participation was not required for this study in accordance with the national legislation and the institutional requirements.

## Author Contributions

Data collection was fulfilled by ZR, JG, and ZZ. Statistical analyses were performed by YX. The manuscript was written by ZR and YX. The study was designed by ZR, JG, and DL. The manuscript was revised by DL, RH, and CH. All authors read and approved the final manuscript.

## Conflict of Interest

The authors declare that the research was conducted in the absence of any commercial or financial relationships that could be construed as a potential conflict of interest.
